# An implementation research programme to support an intravenous iron intervention for pregnant women with moderate and severe anaemia in Malawi: study protocol

**DOI:** 10.1186/s43058-022-00299-x

**Published:** 2022-06-21

**Authors:** Khic-Houy Prang, Elisabeth Mamani-Mategula, Ebony Verbunt, Effie Chipeta, Ricardo Ataide, Martin Mwangi, Kamija Phiri, Sant-Rayn Pasricha, Margaret Kelaher, Lucinda Manda-Taylor

**Affiliations:** 1 Centre for Health Policy, Melbourne School of Population and Global Health, The University of Melbourne, Melbourne, Australia; 2 Kamuzu University of Health Sciences, Blantyre, Malawi; 3 Population Health and Immunity division, Walter and Eliza Hall Institute of Medical Research, Melbourne, Australia; 4 Department of Infectious Diseases, Peter Doherty Institute, University of Melbourne, Melbourne, Australia

**Keywords:** Intravenous iron, Maternal health, Anaemia, Malawi, Low- and middle-income countries, Implementation research

## Abstract

**Background:**

Antenatal iron supplementation is critical to maternal and child health; however, access and adherence to oral iron are inconsistent in many low- and middle-income countries (LMICs). Modern intravenous (IV) iron products have become available in high-income clinical settings and provide an opportunity to deliver high doses of iron in a single-short infusion during pregnancy. However, there is limited knowledge of the drivers and barriers for such an intervention to be effectively delivered and upscaled in LMICs. In this study protocol, we describe the implementation research programme to support an IV iron intervention in Malawi for pregnant women with moderate and severe anaemia.

**Methods:**

The implementation research programme has three phases, each guided by implementation science conceptual frameworks. In Phase 1, we will conduct formative research (context assessment of the health system with key informant interviews) to determine how IV iron can be effectively introduced into routine antenatal care. We will use the findings to co-develop potential strategies with end-users and healthcare providers to improve intervention implementation. In Phase 2, we will disseminate the implementation strategies to support the uptake and delivery of the intervention in the study settings. In Phase 3, the intervention will be implemented, and we will conduct formative evaluation (interviews with end-users, healthcare providers, and analysis of health services data) to investigate the feasibility and acceptability of the intervention and strategies. We will also identify processes and contextual factors that facilitate or impede the delivery and uptake of IV iron.

**Discussion:**

In LMICs, modern IV iron products present a novel opportunity to rapidly cure moderate and severe anaemia in pregnancy, thereby improving maternal and child health outcomes. This implementation research programme will provide guidance and recommendations on how best an IV iron intervention for pregnant women with anaemia can be implemented in an LMIC setting like Malawi. We will develop locally relevant and culturally appropriate implementation strategies by engaging with key stakeholders (pregnant women, healthcare providers, and policymakers) and identifying factors likely to facilitate successful implementation. The findings of this research can guide the implementation of an IV iron intervention in Malawi and other LMICs.

Contributions to the literature

- Modern IV iron products are widely used in high-income countries and provide a novel opportunity to rapidly cure moderate and severe anaemia in pregnancy in LMICs.
- We will co-design implementation strategies with end-users and healthcare workers to inform the delivery of an IV iron intervention in the public healthcare system of Malawi by identifying key modifiable factors at multiple levels (individual, organisation, and health system).
- The findings have the potential to provide a roadmap for policymakers and healthcare organisations on how an IV iron intervention can be effectively delivered and upscaled in Malawi and other LMICs.

## Background

Anaemia in pregnancy remains a significant global health problem, affecting 46% of pregnant women in Africa and 49% in Asia [1]. Antenatal anaemia causes critical risks for both mother and child, increasing maternal mortality from maternal haemorrhage and child mortality from low birth weight and premature delivery [2, 3]. It is estimated that maternal haemorrhage accounts for 3.7 and 12.8% of maternal mortality in Africa and Asia, respectively [4]. Given the prevalence and risks associated with anaemia, in 2012, the World Health Organization (WHO) set a global nutrition target of achieving a 50% reduction of anaemia in women of reproductive age by 2025 [5]. This target was subsequently incorporated as a Sustainable Development Goal indicator in 2020 [6].

In pregnancy, anaemia is commonly due to iron deficiency. Global recommendations for the management of anaemia in pregnancy in low- and middle-income countries (LMICs) advise that women be treated with high-dose daily oral iron (120 mg of elemental iron) supplementation for 3 months [5, 7]. Across sub-Sahara Africa, very few pregnant women receive the recommended course of antenatal iron. Pregnant women commonly present for their initial antenatal visit late in the second trimester, significantly limiting opportunities to treat antenatal anaemia [8].

In Malawi, 33% of women are anaemic, with women more likely to be anaemic when they are pregnant (45%), compared to when they are breastfeeding (30%), or neither pregnant nor breastfeeding (33%) [8]. The government of Malawi provides pregnant women with free daily iron folate supplements each month, regardless of their haematological status or the trimester of pregnancy [9]. However, fewer than 25% of pregnant women receive a full course of antenatal iron [10]. In addition, adherence may be limited by significant gastrointestinal adverse effects [11].

IV iron is increasingly being used for the first-line treatment of iron deficiency in high-income clinical settings, mainly where the need to improve haemoglobin is urgent (e.g., in late pregnancy or before impending major surgery) [12, 13]. Additional benefits of IV iron over oral iron include stronger efficacy (rapid increase in iron stores) and safety, well tolerated with minimal toxicity (limited/no gastrointestinal side effects), and overcoming poor adherence issues commonly associated with oral iron by enabling complete replacement doses in one or two sessions [14, 15].

Modern IV iron products such as Ferric Carboxymaltose (FCM) are available in high-income countries (HICs) and provide an opportunity to give high doses of iron in a single short infusion. Unlike previous IV iron drugs, which required prolonged or multiple infusions and carried a risk of severe reactions, this treatment can be given rapidly, and serious infusion reactions are rare [13, 16]. Despite the benefits of IV iron in rapidly improving iron stores in pregnancy, there have been limited effectiveness trials in LMICs exploring the effects of various IV and oral iron products among pregnant women with anaemia, including hypophosphatemia outcomes in mother and baby. Several randomised controlled trials (RCTs) in Malawi and Nigeria are currently underway to investigate the effectiveness and safety of delivering IV iron compared to standard of care (i.e., oral iron) to pregnant women with anaemia in the second and third trimester on maternal and child health outcomes [17–19].

The impact of IV iron depends on achieving end-users (i.e., pregnant women) access and uptake of this intervention, with support for delivery required from healthcare providers and strong engagement from policymakers. This is a significant challenge given the multitude of actors, components, and processes involved before implementing IV iron in the health system. On average, integrating evidence-based interventions at scale takes 17 years and is likely even longer in lower-resourced settings [20–22]. Implementation research, which seeks to understand what, why, and how interventions work in real-world settings, plays a key role in designing appropriate implementation strategies that bridge the research-to-practice gap [23]. First, understanding the characteristics, behaviours, and needs of the target population and adapting the intervention accordingly to the socio-cultural and institutional contexts are keys to facilitating end-users access and buy-in from healthcare providers [24]. Second, it is important to understand the political and legal landscape that may affect future scale-up of the intervention, such as processes around policy adoption, regulatory approval, human and physical resources, including the capacity of healthcare providers to deliver the intervention, and securing reliable product supply if the intervention is proven to be effective [24].

Despite IV iron being widely available for pregnant women with anaemia in HICs such as Australia and the UK [25], there have been limited studies to explore the acceptability of IV iron among end-users and healthcare providers and to identify both contextual and process factors that may affect the implementation of IV iron intervention across contexts and settings. Several studies reported the following barriers to implementation: cost, the requirement for venipuncture, lack of evidence on safety and efficacy, and shortage of IV iron products. Facilitators to implementation included: avoidance of blood transfusions, addressing noncompliance with oral iron, minimal adverse reactions, reduced risk of postpartum haemorrhage, “champion” healthcare worker, and educating healthcare workers on administration and safety [26–29]. However, all studies focused on the perspectives of healthcare providers, with no end-user perspectives and lacked adequate descriptions of the study settings and the intervention itself (i.e., anaemia detection and delivery of IV iron). Due to the limited implementation research available, it is unclear how an IV iron intervention for pregnant women with anaemia could be effectively delivered in Malawi. Hence, we propose developing, implementing, and evaluating an IV iron intervention for pregnant women with moderate and severe anaemia in routine antenatal care within the public health system in Malawi.

The objective of this paper is to describe the protocol for an implementation research programme which aims to (1) identify health system barriers and enablers to implementing IV iron in routine antenatal care, (2) identify key moments (“touchpoints”) along the patient’s journey that can impact pregnant women’s antenatal care experience to inform the co-development of locally relevant, culturally accepted implementation strategies to support the delivery of the IV iron intervention; (3) assess the acceptability and feasibility of implementing an IV iron intervention for pregnant women with anaemia and healthcare workers, and (4) identify barriers and enablers to delivering and upscaling the IV iron intervention. This is a multi-phase implementation research programme using different methods and approaches to address these aims: (1) formative phase in preparation of IV iron intervention implementation involving reviews, qualitative research, observation, and co-design workshops; (2) dissemination of implementation strategies to support the uptake and delivery of the IV iron intervention; and (3) formative evaluation of the IV iron intervention and strategies involving mixed-methods assessment of the processes and contextual factors affecting its implementation.

## Methods

### Study setting

#### Malawi

**Fig. 1:**
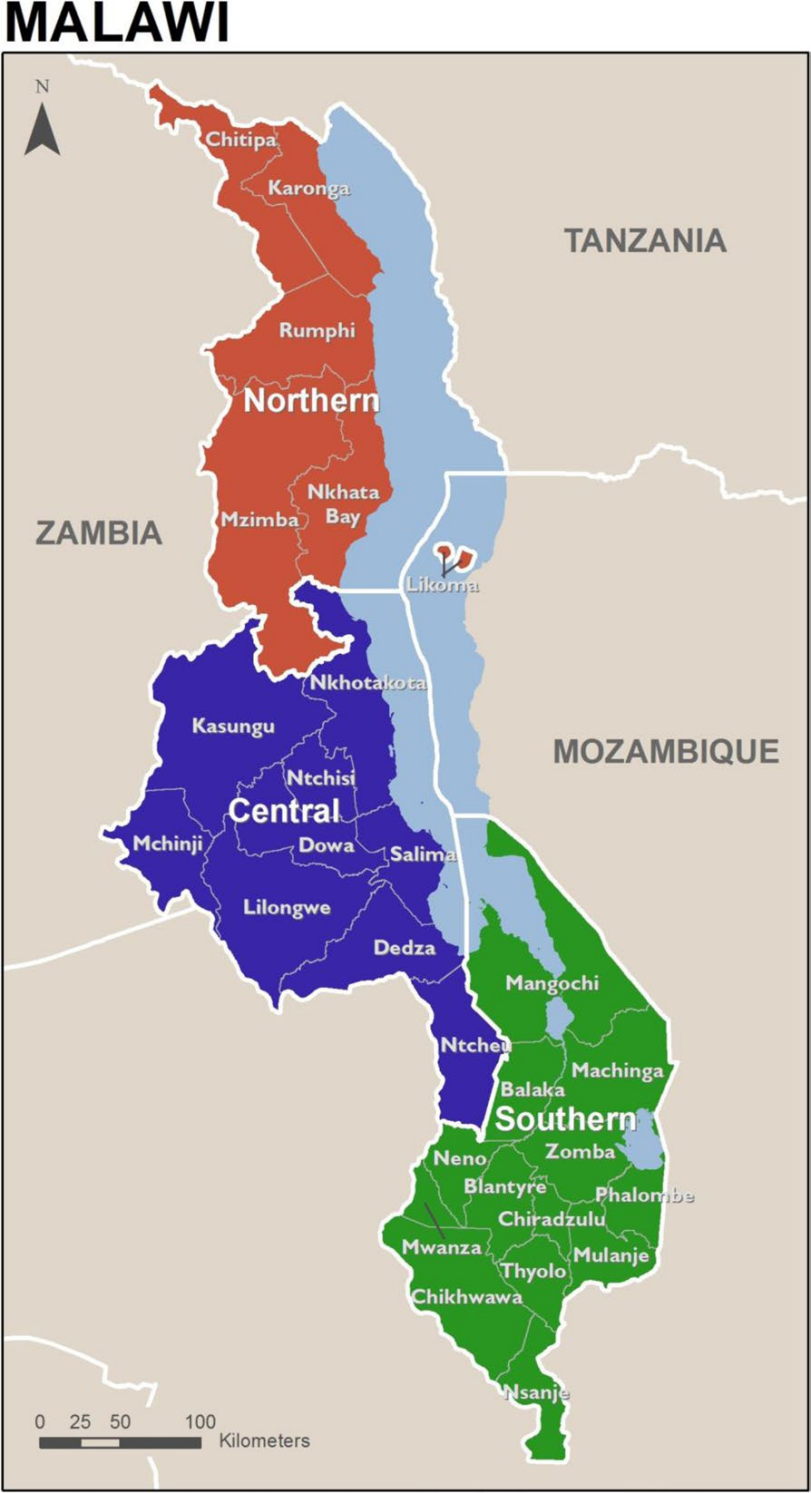
Map of Malawi [8]

We will conduct the study in Malawi in southeast Africa. Malawi shares its borders with Mozambique, Zambia, and Tanzania. As shown in Fig. 1, the country is divided into three regions, which are further divided into 28 districts: Northern (6 districts), Central (9 districts), and Southern (13 districts). In 2019, the country had a population of 18.63 million, with this number expected to double by 2038 [30]. Malawi has a young population, with 70% below 30 years, and only 2.64% aged 65 years and older [31, 32]. Malawi faces a heavy disease burden and is considered malaria-endemic [33]. Malawi has made impressive gains in reducing maternal, neonatal, and child mortality rates, achieving the Millennium Development Goal 4 – Reduce child mortality before the 2015 target date [34]. However, further efforts are required, with Malawi continuing to have one of the highest maternal mortality ratios globally, with an estimated 349 maternal deaths per 100,000 live births [35] and has the highest rate of preterm birth in the world, a leading cause of childhood mortality [36].

#### Zomba district

**Fig. 2:**
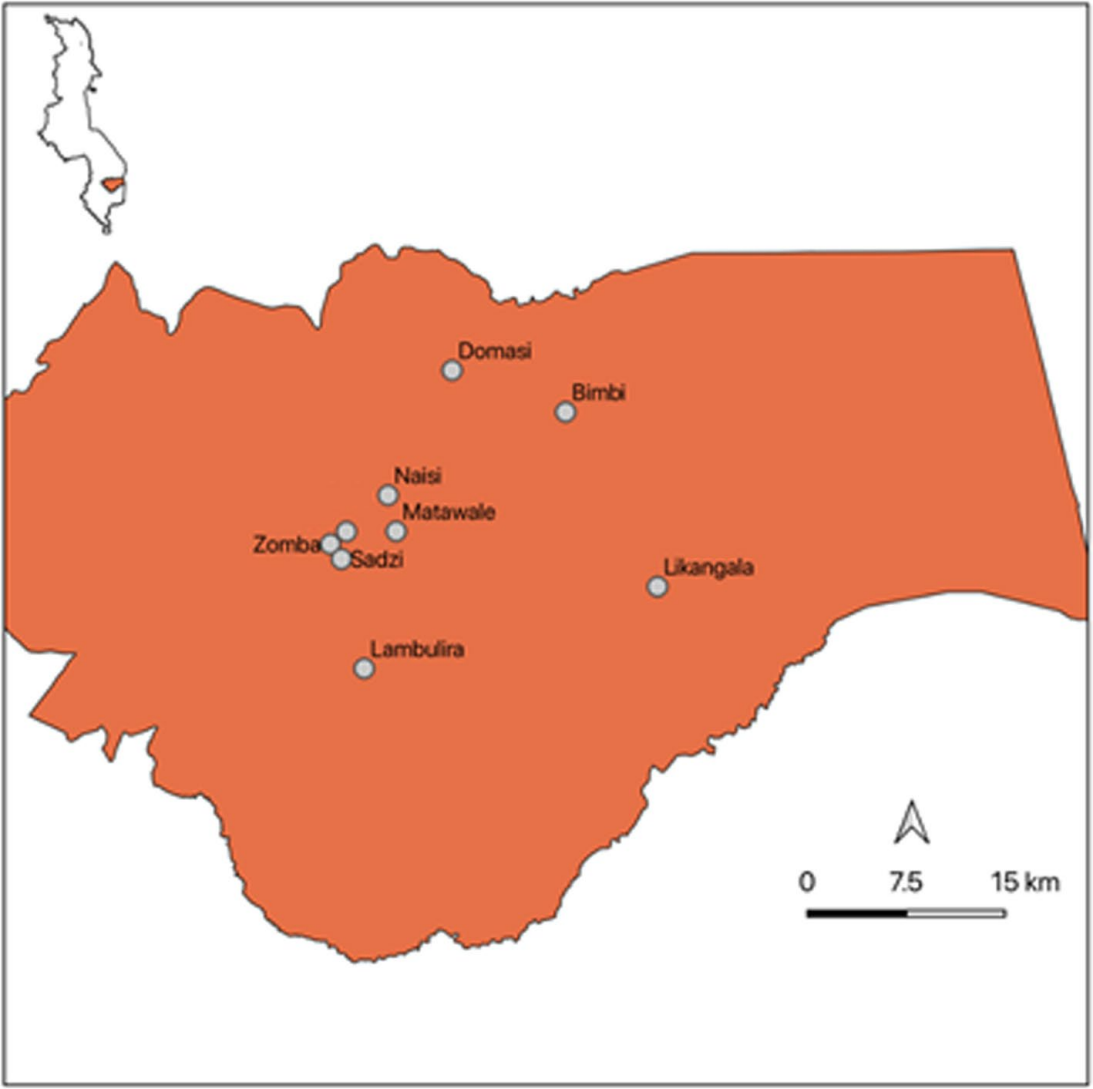
Approximate locations of the health centres in Zomba [37]

We will conduct the study in the Zomba district, situated in the southern region of Malawi. Thirty-seven health facilities serve the district, including health posts and clinics, dispensaries, health centres, and hospitals [37]. The Training Research Unit of Excellence Centre at Zomba Central Hospital in Southern Malawi will manage and oversee the IV iron intervention. The delivery of IV iron intervention for pregnant women in the third trimester with moderate or severe anaemia will operate in health centres across the Zomba district (within a 30-km radius from Zomba): Likangala, Bimbi, Lambulira, Domasi, Naisi, Matawale, City clinic, and Sadzi (Fig. 2). These health centres were selected as they provide antenatal care services to pregnant women in the district.

### Implementation research programme

**Fig. 3:**
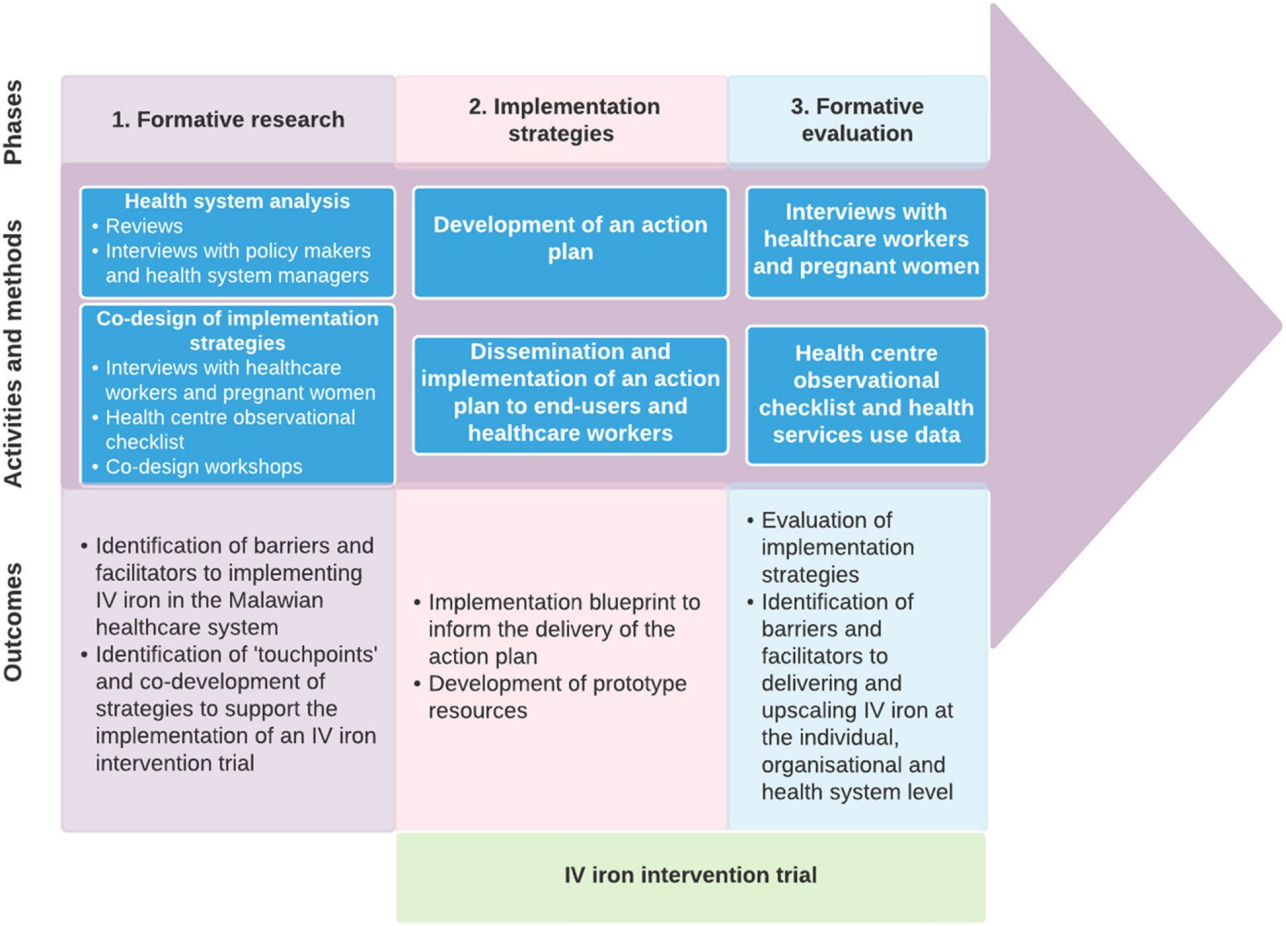
Implementation research design

In this study protocol, we outline the mixed-methods implementation research activities. The implementation research design involves three complementary and partly overlapping phases: (1) formative research including context assessment of the health system, (2) implementation strategies to support the uptake and delivery of the IV iron intervention, and (3) formative evaluation of the intervention processes and implementation strategies (Fig. 3). We describe the formative research and the evaluation of the intervention phases using the Standards for Reporting Implementation Studies (STaRI) checklist [38], with a separate IV iron intervention trial protocol to be reported separately [39].

In Phase 1, the formative research will precede intervention implementation and involves a context assessment of the health system. This will include health system reviews, key informant interviews, and co-design of implementation strategies. As part of the formative research, we will focus on describing how anaemia in pregnancy is currently detected and managed in the study settings and identifying potential barriers and enablers to implementing an IV iron intervention into routine antenatal care within the public health system of Malawi. The findings will then inform the development of implementation strategies to be co-designed with end-users and healthcare providers in a series of workshops to improve the likelihood of the IV iron intervention working in real-world settings.

In Phase 2, an RCT study design will introduce the IV iron intervention at the study sites. The focus of the implementation research will be on implementing an action plan to disseminate the implementation strategies co-developed in Phase 1 to support the uptake and delivery of the IV iron intervention during the trial. The IV iron intervention involves the screening for moderate or severe anaemia (capillary Hb<10g/dl) during the third trimester (27 weeks–35 weeks of gestation) and the administration of IV iron.

In Phase 3, we will conduct a formative evaluation alongside the IV iron intervention trial to explore how the intervention is implemented in real-world settings. We will identify the active components of the IV iron intervention, assess implementation processes and contextual factors, and identify implementation strategies that affect the implementation. We will also explore the acceptability and feasibility of the IV iron intervention among end-users and healthcare providers. This will consist of two components: (1) interviews with pregnant women and healthcare workers and (2) a health centre observational checklist and health services use data. Formative evaluation will enable iterative refinement of the implementation strategies to improve uptake and delivery of the IV iron intervention throughout the trial. We will follow STaRI checklist when reporting the individual implementation research components [38]. In the following sections, we provide a detailed overview of each component for each phase.

#### Conceptual frameworks

We will draw on the Patient-Centred Access to Healthcare (PCAH) [40] and the Consolidated Framework for Implementation Research (CFIR) [41] conceptual frameworks to guide understanding of behaviour change in the implementation context (e.g., uptake of IV iron intervention by pregnant women with anaemia and health centres) and to inform the planning and the implementation of the IV iron intervention. The PCAH defines access as “the opportunity to identify healthcare needs, to seek healthcare services, to reach and obtain/use healthcare services, and to have a need for services fulfilled” [40]. Access results from the interface between the characteristics of the individuals, including his/her social and physical environment (demand-side) and the characteristics of the health systems, organisations, and providers (supply-side). Five dimensions of accessibility capturing supply-side determinants are proposed along the continuum of obtaining care: (1) approachability, (2) acceptability, (3) availability and accommodation, (4) affordability, and (5) appropriateness. Similarly, five corresponding abilities of populations capturing demand-side determinants are proposed along the continuum of obtaining care: (1) ability to perceive, (2) ability to seek, (3) ability to reach, (4) ability to pay, and (5) ability to engage. Although the PCAH does not directly address implementation, it highlights the importance of developing implementation strategies by identifying key modifiable factors along the healthcare continuum, accounting for supply and demand.

The CFIR acknowledges that the effectiveness of interventions implemented within a health system is influenced by the interactions between political, social, and cultural environments and organisational and individual-level factors within the healthcare system [41]. The CFIR organises 39 constructs into five domains: (1) intervention characteristics, (2) outer setting, (3) inner setting, (4) individual (patient/provider characteristics), and (5) process of implementation. The comprehensive and multifaceted nature of the CFIR ensures that the complexities of interventions are captured by explaining “why” implementation succeeded or failed [41]. When used proactively, the CFIR can assist in the identification of relevant modifiable factors that can promote or undermine adoption, implementation, and maintenance [42].

### Phase 1: Formative research including context assessment of the health system

#### Data collection

##### Reviews

In Phase 1, we will conduct two reviews. In the first review, we aim to identify the characteristics of the Malawi health system that impede or facilitate the implementation and upscaling of IV iron into routine care across key dimensions of the health system, including organisational structure, financing, regulation and planning, physical and human resources, provision of services, and principal health care reforms (Appendix 1) [43]. This will also include a grey literature search with documents review of local, state, and national maternal and child health policies programmes and funding structures. We will conduct the first review according to the Preferred Reporting Items for Systematic reviews and Meta-Analyses extension for Scoping Reviews (PRISMA-ScR) checklist [44].

In the second review, we aim to identify processes and contextual factors that have been associated with the effective implementation of IV iron intervention for pregnant women with anaemia in HICs and LMICs. This will be followed by assessing the applicability and transferability of IV iron intervention using an appraisal tool [24] (Appendix 2). Based on the IV iron intervention knowledge derived from both reviews, we will develop a list of attributes that may impact applicability and transferability. Finally, we will rate the applicability and transferability of the intervention to the local setting. We will specify in advance the objectives, inclusion criteria, and review methods and document it in a protocol. We will conduct the second review following the Preferred Reporting Items for Systematic Reviews and Meta-Analyses (PRISMA) [45].

##### Key informant interviews

To complement the reviews, we will conduct semi-structured interviews (~45–60 min) with key informants, including policymakers from the Ministry of Health and District health system managers from the Zomba district (*n*=20), to identify constraints and resources available and the most promising strategies for incorporating IV iron into the health system and the support required to do this. Ministry of Health employees to be interviewed may include the Chief Pharmacist, Principal Secretary of Health, Director of Medical Services, Director of Planning and Policy Development, Director of Preventive Health Services, Director of Reproductive Health Services, and Director of Research. District health system managers to be interviewed may include the district health/nursing officers, district Safe Motherhood coordinators, facility administrators, and personnel responsible for maternity activities in each of the eight health centres in the Zomba district. We will approach policymakers and health system managers via email or telephone and invite them to be interviewed. We may use snowballing sampling in which participants may be asked to identify and refer colleagues that would be a good fit for the research. After completing each semi-structured interview, we will invite participants who appear to be interested in providing more feedback to participating in a consultation workshop.

We will develop an interview guide for policymakers and health system managers informed by the PCAH and will focus on the following areas to support the delivery and upscaling of IV iron in routine antenatal care: Malawian health system with a focus on antenatal care delivery, antenatal care delivery strengths and areas for improvement, identify levers for implementing change (including key stakeholders and policy processes), potential barriers and enablers associated with the implementation of IV iron, anticipated unintended consequences arising from the implementation of IV iron, and processes of upscaling IV iron in the health system. In addition, we will triangulate data from the database search, grey literature search, and data from semi-structured interviews conducted with policymakers, government partners, and health system managers to explore the factors influencing anaemia detection and management and potentially influencing IV iron intervention implementation.

##### Co-design of implementation strategies

We will adopt a co-design approach to develop implementation strategies to support the uptake and delivery of IV iron. Co-design is a participatory approach to developing implementation interventions or strategies that bring together healthcare providers and patient experience to design solutions to problems, ensuring that solutions are developed to understand the local context and that the result meets all stakeholders’ needs [46]. Co-design is a two-staged approach that typically involves an information-gathering stage followed by a second stage in which end-users jointly develop the intervention or strategies with healthcare providers and the research team. The information-gathering stage identifies the “touchpoints” (i.e., positive/negative contacts the end-users have with healthcare services which could be improved) and further develops an in-depth understanding of these with end-users, healthcare providers, and other stakeholders. This will include interviews with healthcare workers, pregnant women, and a health centre observational checklist. The co-design stage will involve established groups working together to co-develop solutions to the identified areas for improvement through a series of structured and facilitated workshops.

##### Interviews with healthcare workers

We will conduct semi-structured interviews with healthcare workers (*n*=32; 4 per site) employed across one of the eight sites in the Zomba district. Healthcare workers may include doctors, nurses, allied health professionals, and support staff. A list of healthcare workers involved in the trial will be provided to us by the clinical trial coordinator. We will contact each healthcare worker via email or telephone and invite them to participate in the interviews.

We will develop an interview guide for healthcare workers informed by the CFIR framework and will focus on identifying the touchpoints to support the implementation of IV iron intervention, which will be further discussed in the co-design workshops: role of healthcare worker, access to facilities for appropriate implementation, healthcare worker training (e.g., processes to improve the identification of eligible women and to screen for anaemia, administration of IV iron and safety monitoring), logistics of health service delivery (e.g., integration of IV iron administration with other health care), delivery supply chain, medical record keeping, and support for women’s participation.

##### Interviews with pregnant women

We will also draw on the findings from a previous qualitative study with community members describing the opinions and experiences on the acceptability of IV iron to treat anaemia in pregnancy in Malawi (unpublished data) as part of a previous trial investigating the effectiveness of IV iron on anaemia in pregnant women in the second trimester [18, 19]. Fifteen in-depth interviews and two focus group discussions with pregnant women and seven in-depth interviews with healthcare workers at a community-based health centre in Blantyre and a tertiary hospital in Zomba were conducted. For pregnant women, the interview guide and focus group discussion focused on concepts, procedures, experiences, and health outcomes, whilst for healthcare workers, the emphasis was on perceptions about IV iron treatment and barriers and enablers to implementing IV iron treatment. The interviews and focus group discussions with participants were audio-recorded and conducted in private rooms at the health centres. Interviews and focus group discussions with pregnant women were conducted in Chichewa, and the interviews with healthcare workers were conducted in English.

##### Co-design workshops

We will conduct several co-design workshops with healthcare workers and community members (i.e., a person residing within the local health service catchment area including those with a formal leadership role in the community (e.g., village chief), women within the targeted sample age group visiting the health centre, and mothers and partners of pregnant women who attend the health centre) to co-develop solutions to improve the implementation of IV iron based on the “touchpoints” identified in the semi-structured interviews. The workshops will include (1) an understanding of the patient’s journey (i.e., pregnant women) through the healthcare system, (2) a review of existing service processes and the identification of areas for improvement related to the touchpoint in question, and (3) a review of good practice examples and discussion of the ideas for action plans.

### Phase 2: Implementation strategies to support the uptake and delivery of IV iron intervention

#### Development of an action plan

We will develop an action plan to address the key touchpoints raised following the co-design workshops. In the action plan, we will focus on the strategies co-developed during the workshops to improve the delivery of IV iron intervention for both end-users and healthcare workers at the health centre and health system levels: (1) pre-implementation including recruitment of eligible pregnant women and (2) implementation, including identifying and screening eligible pregnant women for anaemia and administration of IV iron. We will map the action plan to the CFIR [41] and Action, Actor, Context, Target, and Time (AACTT) framework [47] to specifically describe the behaviours of multiple actors across different levels of the organisation required to enact change. We will distribute the action plan to the collaboration group (consisting of the implementation science and trial research teams) for comments and further discussion. The collaboration group will review, prioritise, and refine the strategies identified to be implemented based on empirical evidence and pragmatic rationales such as feasibility and resource capacity.

#### Dissemination and implementation of an action plan

We will finalise the implementation strategies and detailed them in an implementation blueprint to inform the delivery of the IV iron intervention trial. The selected strategies to be implemented will aim to support specific aspects of the trial. For example, potential strategies derived from co-design workshops may include additional end-user educational resources to alleviate IV iron misconceptions and raise community awareness about the importance of iron for maternal and child health, regardless of treatment modality. The educational resources may be disseminated via outreach groups such as district environmental health officers and health service assistants to the community. We will disseminate the strategies before trial commencement at each site and throughout the trial. We will support the trialists to deliver the implementation strategies selected. In addition, issues and strategies identified beyond the scope of the IV iron intervention (e.g., transportation to the health centre, limited access to maternal and child health services, workforce capacity) will be disseminated and discussed with policymakers such as the Safe Motherhood Technical Working Group in an external consultation meeting, with the primary focus on integrating and upscaling of IV iron intervention in routine antenatal care if the trial is successful.

### Phase 3: Formative evaluation of implementation IV iron intervention and strategies

In Phase 3, we will evaluate the implementation of the intervention, including its strategies, using a formative evaluation approach. Formative evaluation is “a rigorous assessment process designed to identify potential and actual influences on the progress and effectiveness of implementation efforts” in complex or uncertain environments [48]. We will use implementation and progress-focused formative evaluation to identify processes and contextual factors that facilitate or impede the delivery and uptake of IV iron and assess various implementation outcomes among pregnant women and healthcare workers. This will enable us to identify challenges that may have emerged during the implementation process and provide feedback to the trial research team and healthcare workers to adapt and improve the process, thus facilitating a continuous development loop. The implementation and progress-focused formative evaluation will involve an iterative approach where periodic reporting cycles (e.g., 6 months) will reinforce progress via positive feedback or inform refinements to the intervention implementation processes and implementation strategies. This will involve consultations with key stakeholders to enhance existing strategies or co-design additional strategies, which will, in turn, be evaluated after the intervention using an interpretive formative evaluation. Interpretive formative evaluation can help determine whether the IV iron intervention was successfully implemented, including providing learnings both within and between health centres given the multicentre focus of the IV iron intervention, understanding the impact of the implementation strategies, and identifying the barriers that need further attention before widespread scale-up.

#### Conceptual frameworks

We will draw on the PCAH [40] (as described above) and the Implementation Outcomes framework (IOF) [49] to evaluate the IV iron intervention implementation and strategies. IOF defines implementation outcomes as “the effects of deliberate and purposive actions to implement new treatments, practices, and services” [49]. Implementation outcomes have three functions: (1) serve as indicators of the implementation success, (2) serve as proximal indicators of implementation processes, and (3) act as key intermediate outcomes about service system or clinical outcomes in treatment effectiveness and quality of care research. The core set of implementation outcomes are (1) acceptability, (2) adoption (also referred to as “uptake”), (3) appropriateness, (4) feasibility, (5) fidelity, (6) implementation cost, (7) penetration, and (8) sustainability.

#### Data collection

##### Interviews with healthcare workers and pregnant women

We will conduct semi-structured interviews (~45–60 min) with healthcare workers (*n*=32; 4 per site) delivering the IV iron intervention and pregnant women with anaemia (*n*=40; 5 per site) who are enrolled in the IV iron intervention. A list of healthcare workers involved in the IV iron intervention trial will be provided by the clinical trial coordinator to us. We will contact each healthcare worker via email or telephone and invite them to interview. Pregnant women enrolled in the trial will be asked by the clinical trial coordinator whether they would like to be interviewed about their experiences in the trial and consent for their contact details to be passed on to us. If they consent, the clinical trial coordinator will then provide a list of pregnant women enrolled in the trial and we will contact each woman via telephone and invite them to participate in an interview.

Specific formative evaluation measures are described in Table 1. For pregnant women, we will focus on identifying demand-side determinants from the PCAH that impede or facilitate access to and uptake of IV iron, including referrals to the health centre to receive IV iron, acceptability of receiving IV iron, participation in ongoing care, and satisfaction with care. For healthcare workers, we will focus on identifying the barriers and enablers across the stages of the implementation of the intervention that they are involved in using the IOF: access to ongoing training and support, staff confidence in implementing IV iron (and similar interventions), effectiveness and efficiency of implementation, barriers and enablers to implementation, improvements/problems in implementation since the last interview, unintended consequences of implementation, and strategies to improve implementation.

**Table 1:** Formative evaluation measures and corresponding conceptual framework domains

Implementation outcomes	Frameworks	Measures	Data source
Acceptability	Implementation Outcomes Framework	• Acceptability of anaemia screening by healthcare workers/pregnant women • Acceptability of providing IV iron by healthcare workers/women	• Interviews with healthcare workers and pregnant women
	Patient-Centred Access to Healthcare – Demand side	• Cultural and social factors make it possible for pregnant women to accept these services, and their use of the services is seen as appropriate	• Interviews with pregnant women
Appropriateness	Implementation Outcomes Framework	• Anaemia screening meets the need of healthcare workers /pregnant women • IV iron intervention meets the need of healthcare workers/pregnant women	• Interviews with healthcare workers and pregnant women
	Patient-Centred Access to Health care – Demand side	• Fit between the service and pregnant women’s need, its timeliness, the amount of care spent in assessing health problems and determining the correct treatment and the technical and interpersonal quality of the services provided	• Interviews with pregnant women
Adoption	Implementation Outcomes Framework	•Uptake of anaemia screening by healthcare workers/pregnant women •Uptake of IV iron intervention by health care workers/pregnant women	• Health services use data
Feasibility	Implementation Outcomes Framework	• Facilitators for anaemia screening • Facilitators for IV iron intervention • Strategies to improve anaemia screening and IV iron intervention	• Interviews with healthcare workers • Health centre observational checklist
Fidelity	Implementation Outcomes Framework	• Anaemia screening and IV iron intervention implemented as planned • Strategies to support the implementation	• Interviews with healthcare workers • Health services use data • Observational checklist of anaemia screening and IV iron intervention
Implementation cost	Implementation Outcomes framework	• Cost of healthcare resources to deliver the IV iron intervention • Cost of resources to develop and execute the implementation strategies	• Interviews with healthcare workers • Financial records from implementation science and trial research team, and health centres
Penetration	Implementation Outcomes framework	• Reach of the screening programme for pregnant women • Strategies to increase reach • Effectiveness of follow-up measures in reaching pregnant women who needed IV iron	• Interviews with healthcare workers • Health services use data
Sustainability	Implementation Outcomes framework	• Capacity to sustain anaemia screening and IV iron intervention • Strategies to improve sustainability	• Interviews with healthcare workers
Approachability	Patient-Centred Access to Health care – Demand side	• Pregnant women with health needs can identify that antenatal screening and associated treatments exist, can be reached, and may have a positive impact on their health	• Interviews with pregnant women • Health services use data
Availability and accommodation	Patient-Centred Access to Health care – Demand side	• Health services (either the physical space or those working in health care roles) can be reached both physically and promptly	• Interviews with pregnant women • Health services observational checklist
Affordability	Patient-Centred Access to Health care – Demand side	• Economic capacity for pregnant women to spend resources and time to use services	• Interviews with pregnant women

##### Health centre observation visits and health services use data

We will conduct health centre observation visits using a structured checklist based on an adaptation of the WHO service availability and readiness assessment tool [50] and collect health services use data throughout the formative evaluation of the intervention. In the health centre observation visits, we will focus on describing the characteristics of the health centre and the detection and management of anaemia in pregnancy, including how the healthcare workers are identifying, screening, and treating anaemia (Table 2). Using the health services data, we will focus on describing the number of pregnant women with anaemia who presented to the health centre for treatment across the stages of the implementation of the intervention (Table 3). In addition, we will request aggregated de-identified health services use data from each health centre participating in the trial.

**Table 2:** Observational checklist measures

	Measures
**Infrastructure including diagnostic capacity**	Condition of facilities • Dedicated waiting room • Private consultation room • Available hygienic space to deliver IV iron
	Access to pathology laboratories • On-site • Off-site
**Services including equipment**	Staff training • Identification • Screening • Diagnosis/tests • Treatment • Education and counselling • Audit and monitoring
**Staffing**	Staffing for screening, administration, and safety monitoring • Roles and responsibilities of healthcare workers
	Quality of record-keeping • Medical record system • Data collection • Audit and monitoring • Women’s health passport
**Medicines and commodities**	Supply chain and availability of consumables • Forecasting • Procurement • Distribution • Storage • Disposal

**Table 3:** Health services use measures

Stage of the intervention	Measures
Identification of eligible women	• Number of eligible pregnant women attending health centre • Date eligibility ascertained for each woman
Screening for anaemia and other conditions	• The number of pregnant women screened for anaemia on the same day as eligibility determined • Number of pregnant women screened for anaemia on subsequent visits • The number of pregnant women screened for other conditions on the same day as eligibility determined • Number of pregnant women screened for anaemia on subsequent visits • Number of pregnant women screened for other conditions on subsequent visits • Number of pregnant women with anaemia • Number of pregnant women with other conditions • Number of pregnant women with contraindications for IV iron
Administration of IV iron	• Number of pregnant women excluded because of contraindications • Number of pregnant women where administration delayed pending further medical advice • Number of pregnant women administered IV iron on the same day as the screening • Number of pregnant women screened administered IV iron on subsequent visits
Monitoring safety following IV iron	• Number of pregnant women monitored following IV iron administration • Number of safety incidents • Actions following safety incidents
Referral other conditions	• Number of pregnant women referred for other conditions
Treatment other conditions	• Number of pregnant women treated for other conditions
Ongoing access to health services	• Number of health centre visits • Number of pregnant women completing an appropriate cycle of antenatal care • Type of delivery • Place of delivery

### Data analysis plan

#### Interviews

Past qualitative research has shown that saturation of themes is likely to be achieved with approximately 12–20 interviews in the homogenous group [51, 52]. As such, we require a minimum of 10 participants for each qualitative study to ensure that there is sufficient data to allow for qualitative assessment of key themes. For healthcare workers and pregnant women, we will aim to recruit a minimum 4 to 5 participants per site to identify processes and contextual factors influencing implementation of the intervention and implementation outcomes that may differ across sites.

We will audio-record and transcribe all interviews for thematic analysis. We will enter and code into NVivo 12 to ensure data integrity and enhance analysis. We will cross-check all transcriptions to ensure validity. The methodological approach to analysing interview data will be iterative, within and between participants. Data iteration will introduce new topics raised by participants and enable the identification of new patterns and emerging themes. We will use a combination of inductive and deductive coding for identifying, analysing, and reporting patterns within the data [53, 54]. Two research team members will independently analyse a subset of interview transcripts using a coding tree developed from the structure of the interview questions. We will then compare and refine the resulting coding trees (theme lists) through discussion between us, developing an agreed coding tree. One researcher will then code the remaining interview transcripts, and emergent themes will be added as needed. For theme development and revision, we will cluster similar codes together and subsequently collapsed into emergent themes and mapped to the CFIR domains and constructs where applicable [41]. We will report the qualitative studies using the Standards for Reporting Qualitative Research reporting guideline [55].

#### Co-design workshops

For each co-design workshop, we will invite a maximum of 20 participants. Previous case studies and review have reported between 2 and 16 participants per co-design workshop [56, 57]. A number larger than this may not allow all participants to actively participate and small workshops are considered to be more comfortable for participants.

We will audio-record and take notes during the co-design workshops. We will collate and summarise participants’ notes from group activities following each workshop. We will provide the findings from each workshop back to participants for feedback. Data from these workshops will inform the development of implementation strategies to improve the uptake and delivery of IV iron intervention based on the touchpoints identified in the interviews.

#### Health centre observation visits and health services use data

We will conduct descriptive analyses using Stata for the health centre observation visits and health services use data. As the implementation research progresses, we will compare the baseline data and subsequent rounds of data collection using inferential statistics to compare pre-intervention to post-intervention for each measure. Health centre observation visits and health service use data will enable a better understanding of how healthcare workers work with pregnant women eligible for the intervention. We will triangulate qualitative and quantitative data to understand how implementation can be strengthened across the trial and within each site.

### Ethics

We obtained ethics approval for the implementation research from the Melbourne School of Population and Global Health Human Ethics Advisory Group, The University of Melbourne, and the College of Medicine Research and Ethics Committee, The University of Malawi. We will seek informed consent from all participants before participating in the implementation research activities. The consent forms and interview guides are available in Chichewa and English.

### Compensation and reimbursement to study participants

We will compensate participants in both the qualitative research and co-design research activities for their time US$10 (~MWK $7338.01) and reimbursed for their travel costs to the venue. We will provide participants with refreshments, including morning and afternoon tea and lunch during the co-design workshops. Through the National Health Sciences Research Committee, the Malawi government recommended that all human subjects research offer participants US$10 per study visit as compensation for costs [58, 59]. Given their roles and responsibilities, we will not offer policymakers and health system managers compensation to participate in the semi-structured interviews.

### Study timeline

The timeframe for the implementation research programme (Phases 1, 2, and 3) is approximately 4 years. When this paper was submitted for publication, Phase 1 (formative research) was underway and will be completed in 2022. Phase 2 (implementation strategies) will occur before the rollout and throughout the IV iron intervention trial. Phase 3 (formative evaluation) will be ongoing throughout the implementation of the IV iron trial, which is anticipated to be over 12 months. Report writing and results dissemination will occur following the completion of each phase.

## Discussion

Globally, antenatal anaemia continues to pose serious risks to both mother and child [1–3]. Iron supplementation to infants has limited benefit on functional outcomes [60], potentially raising the priority for anaemia control in pregnancy. Given that IV iron has been demonstrated to be an effective and safe treatment in HICs for pregnant women with moderate and severe anaemia [14, 15, 61], there is an opportunity to adopt this vital intervention to LMICs, such as Malawi, to ensure equitable access to health care [23]. Implementation research plays a key role in supporting the uptake and delivery of IV iron intervention in routine care by ensuring that the intervention is applicable to LMICs and transferable to their end-users and healthcare providers from HICs [23]. In this paper, we describe the protocol for an implementation research program, which aims to support the implementation of an IV iron intervention for pregnant women with moderate and severe anaemia in routine antenatal care within the public health system in Malawi.

### Expected study outcomes

The main outcomes of this implementation research programme will include a thorough understanding of (1) contextual factors, including constraints and resources available to support the implementation of an IV iron intervention in the Malawi public health system and (2) locally relevant and culturally appropriate implementation strategies that support the uptake and delivery of an IV iron intervention in countries with comparable health care systems. Overall, these findings will contribute to the growing body of evidence and provide a roadmap for policymakers and healthcare organisations on how modern IV iron products can be safely delivered to pregnant women with anaemia by the public health system in a low-resource setting as Malawi.

### Anticipated problems and solutions

The implementation research will be implemented during COVID-19 times. We will adhere to the Malawian’s public health guidelines. The research will be managed and coordinated to ensure safety and minimise risks to participants and researchers. A contingency plan to address the potential impacts of COVID-19 will be undertaken and reviewed as required. This will include an assessment of the potential data collection risks associated with COVID-19 transmission by the severity of harm and likelihood of COVID-19 transmission. This may mean that interviews are conducted over Zoom or telephone instead of face-to-face.

### Applicability of the results

Health care systems in LMICs work under increasingly dynamic and resource-constrained conditions. Evidence-based strategies are essential to ensure that research investments maximise healthcare value and improve public health [62]. Our implementation research programme will develop such evidence-based strategies to inform the implementation of an IV iron trial—increasing the likelihood of the intervention being delivered as intended—and therefore improving the internal validity of the trial findings. From the outset, this implementation research programme uses multiple approaches involving qualitative and quantitative methods, including engagements and participation with policymakers, healthcare providers, and end-users to maximise the scalability of our findings. The results of our formative research will identify contextual factors, including constraints and resources available to support the implementation of an IV iron intervention, that, if successful, could facilitate scale-up in Malawi and development and implementation in other LMICs.

### Dissemination of findings

We will disseminate the findings from this implementation research programme to key stakeholders through several outputs, including regular presentations to specific interest groups (such as the Safe Motherhood Technical Working group in Malawi), evidence briefs, meetings with community members, and journal articles. We will also share results at relevant conferences.

## Data Availability

Not applicable

## Appendix 1

**Table 4:** Key dimensions of a health system [43]

Dimensions	Description
Organisational structure	provides an overview of how the health system in the country is organised and outlines the main actors and their decision-making powers; discusses the historical background for the system; and describes the level of patient empowerment in the areas of information, rights, choice, complaints procedures, safety, and involvement.
Financing	provides information on the level of expenditure, on who is covered, which benefits are covered, the sources of health care finance, how resources are pooled and allocated, the main areas of expenditure, and how providers are paid.
Regulation and planning	addresses the process of policy development, establishing goals and priorities; deals with questions about relationships between institutional actors, with specific emphasis on their role in regulation and what aspects are subject to regulation; and describes the process of health technology assessment (HTA) and research and development.
Physical and human resources	deals with the planning and distribution of infrastructure and capital stock; the context in which IT systems operate; and human resource input into the health system, including information on registration, training, trends, and career paths.
Provision of services	concentrates on patient flows, organisation and delivery of services, addressing public health, primary and secondary health care, emergency and daycare, rehabilitation, pharmaceutical care, long-term care, services for informal caregivers, palliative care, mental health care, dental care, complementary and alternative medicine, and health care for specific populations.
Principal health care reforms	review reforms, policies, and organisational changes, which have substantially impacted health care.

## Appendix 2

**Table 5:** Attributes of applicability and transferability of the intervention [24]

	**Questions**
**Applicability**	
Political environment	Does the political environment of the local society allow this intervention to be implemented? Is there any political barrier to implementing this intervention?
Social acceptability	Would the general public and the targeted (sub)population accept this intervention? Does any aspect of the intervention go against the local social norms? Is it ethically acceptable?
Cultural adaptability	Can the contents of the intervention be tailored to suit the local culture?
Resource implications	Are the essential resources for implementing this intervention available in the local setting?
Educational level of the target population	Does the target population in the local setting have a sufficient educational level to comprehend the contents of the intervention?
Organisational structure	Which organisation will be responsible for the provision of this intervention in the local setting? Is there any possible barrier to implementing this intervention due to the structure of that organisation?
Skills of local interventionists	Does the provider of the intervention in the local setting have the skill to deliver this intervention? If not, will the training be available?
**Transferability**	
Baseline prevalence of risk behaviours/infection	What is the baseline prevalence of the health problem of interest in the local setting? What is the difference in prevalence between the study setting and the local setting?
The characteristics of the target population	Are the characteristics of the target population comparable between the study setting and the local setting? Regarding the particular aspects that will be addressed in the intervention, is it possible that the characteristics of the target population (e.g. ethnicity, socioeconomic status, educational level) will impact the effectiveness of the intervention?
The capacity to implement the intervention	Is the capacity to implement the intervention comparable between the study setting and the local setting in such matters as the political environment, social acceptability, resources, organisational structure, and the local providers’ skills?

## Abbreviations

AACTT: Actor Action Context Target Time
IV iron: Intravenous iron
IOF: Implementation Outcomes Framework
LMIC: Low- and middle-income country
HICs: High-income country
WHO: World Health Organization
FCM: Ferric carboxymaltose
RCT: Randomised controlled trial
StaRI: Standards for Reporting Implementation studies
PCAH: Patient-Centred Access to Healthcare
CFIR: Consolidated Framework for Implementation Research
PRISMA: Preferred Reporting Items for Systematic Reviews and Meta-Analyses
PRISMA-SCR: Preferred Reporting Items for Systematic Reviews and Meta-Analyses extension for SCoping Reviews
